# Playing the Self, Paying the Price: A Dual‐Path Model of Avatar Identification and Online Game Consumption

**DOI:** 10.1002/pchj.70125

**Published:** 2026-10-15

**Authors:** Yuchen Yang, Su Tao, Dong Wei, Mengdie Yang, Jinghan Liang

**Affiliations:** ^1^ China University of Geosciences (Beijing) Beijing China

**Keywords:** avatar identification, character competence value, online game consumption, perceived enjoyment, visual authority value

## Abstract

With the rapid expansion of the online gaming market, understanding the psychological mechanisms underlying player consumption behavior has become increasingly important. This study investigates how online game players' identification with virtual avatars is associated with perceived consumption value and, in turn, with gaming experience and in‐game spending. Two substudies were conducted with a total of 669 participants. Study 1 employed a questionnaire‐based survey to examine the relationships among core variables. Study 2 targeted players of *Honor of Kings*, a widely popular online game, to further test the proposed mediation model. The findings indicate that avatar identification significantly predicts perceived enjoyment through two mediators, specifically visual authority value and character competence value, which in turn are associated with players' consumption behaviors. Furthermore, the association between identification with a specific avatar and consumption intention provides additional support for the proposed mediation pathways. This research contributes to an “Identification–Value–Experience” framework for understanding online game consumption, suggesting that avatars serve not only as extensions of the self but also as sources of perceived value relevant to enjoyable gaming experiences and consumer decision‐making. Practical implications are discussed for game developers aiming to optimize user experience and inform monetization strategies.

## 1 Introduction

With the rapid development of the internet, online game consumption (OGC) has become an increasingly prominent component of overall online consumer behavior (Hamari and Keronen 2017). According to the 2024 Global Games Market Report released by Newzoo, the global gaming market is projected to reach USD 187.7 billion in 2024, reflecting a 2.1% year‐over‐year growth and setting a new record high (Newzoo 2024). Some game‐related features, such as service quality and customization options (Guo and Barnes 2012), have been shown to significantly influence players' spending behavior. Among these, customization has emerged as a particularly impactful factor, largely due to the high degree of autonomy it offers players (Teng 2010). In online games, players can freely modify their avatar's appearance to reflect personal preferences, including choices in outfits, hairstyles, and equipment. This process of “avatar customization” not only results in the creation of a unique virtual character but also facilitates the projection of the player's ideal self‐image and personal values onto the avatar (Ducheneaut et al. 2009). In doing so, it fosters a psychological bond known as avatar identification (Van Looy et al. 2012).

Avatar identification refers to a temporary shift in self‐perception resulting from the integration of an individual's self‐concept with a virtual character (Van Looy et al. 2012). This psychological construct has been strongly associated with behavioral outcomes such as online game engagement (Li et al. 2024) and Internet gaming disorder (Yang et al. 2025; Zhang et al. 2024). However, research directly examining the relationship between avatar identification and consumption behavior remains limited. One notable study found that identification with luxury avatars was positively associated with players' willingness to purchase luxury virtual goods (Sharma 2024). Nevertheless, status‐driven consumption of luxury fashion items represents only a narrow subset of in‐game spending. OGC also includes a wide range of functional and hedonic purchases (Hamari 2010). Luxury avatar identification typically reflects a form of identity expression rooted in social comparison and conspicuous consumption (Sharma 2024; Teng 2017). In contrast, the more commonly studied form of avatar identification is grounded in perceived character similarity and self‐extension, representing a more personal and generalized sense of virtual identity (İskender 2023; Teng 2017; Van Looy et al. 2012). Whether this form of avatar identification is associated with consumption beyond status‐oriented purchases remains unclear.

Accordingly, the present study draws on the Theory of Consumption Values (Sheth et al. 1991) to examine the psychological processes underlying the association between avatar identification and OGC. Specifically, we focus on two forms of avatar‐related consumption value, visual authority value and character competence value, and examine the role of perceived enjoyment in linking these value perceptions to consumption‐related outcomes. Two core questions are addressed: (1) Is avatar identification associated with players' willingness to spend and actual in‐game expenditures? (2) What types of value perceptions and psychological experiences are associated with avatar identification and OGC? Addressing these questions contributes to a deeper understanding of the psychological foundations underlying the virtual identity economy. Moreover, the findings offer practical insights for the design of game products and the development of targeted marketing strategies.

### 1.1 Avatar Identification and OGC

Avatar identification may contribute to increased spending in online games. On the one hand, players who strongly identify with their avatar tend to form deeper emotional bonds with their virtual characters, experience deeper immersion (Zhang et al. 2024), and exhibit greater engagement and participation during gameplay (Li et al. 2024). Prior studies have shown that engagement is closely linked to OGC behaviors (Blohm and Leimeister 2013; Naureen and Faiz 2026). On the other hand, drawing on self‐congruity theory, research has demonstrated that consumers are more inclined to purchase products that help them approach their ideal self, as such purchases fulfill self‐esteem needs and promote self‐actualization (Sirgy 2018). This theoretical framework can be extended to the online gaming contexts. Many players view their avatars as extensions of the self (Bessière et al. 2007), using them as tools for communication and self‐expression. Through feedback from others, such as social evaluations and peer attitudes, players continuously reconstruct their self‐concept (Cooley 2017). Within this framework, in‐game consumption that enhances an avatar's status or abilities may be perceived as instrumental in achieving one's ideal self (Sharma 2024).

Empirical findings lend further support to these theoretical propositions. For instance, players often purchase virtual items for their avatars as a means of compensating for real‐life deficiencies, thereby creating a more idealized version of themselves within the game (Kim et al. 2012). Moreover, the closer an avatar resembles a player's ideal self, the more likely the player is to develop attachment to the character, which subsequently increases consumption intentions (Duan and Jeong 2025; Wang et al. 2024). Taken together, these findings suggest that the pursuit of the ideal self, facilitated by avatar identification, may be associated with both immersive engagement and in‐game consumption (Naureen and Faiz 2026). Accordingly, this study proposes the following hypothesis:

*Avatar identification is positively associated with online game consumption*.

### 1.2 Avatar Identification, Perceived Enjoyment, and OGC

If H1 is supported, a subsequent question arises: What kind of psychological experiences may account for the relationship? Perceived enjoyment may serve as a bridge between strong avatar identification and increased OGC. Previous studies have consistently shown that the more individuals identify with their avatars, the greater the enjoyment they experience during gameplay (Böffel et al. 2022). Social interaction through avatars has been shown to enhance this sense of enjoyment (Gu et al. 2025), and avatar customization significantly boosts players' positive emotions (Kang and Kim 2020). Players who strongly identify with their avatars tend to derive more enjoyment from the gaming experience (Lee et al. 2023).

Several psychological processes may help explain this association. First, higher avatar identification allows players to project their own emotions and cognitions onto the virtual avatar (Wang et al. 2024), enhancing emotional investment and the perceived authenticity of the virtual world, both of which increase immersion and enjoyment (İskender 2023; Zhang et al. 2024). In addition, avatar identification is positively associated with embodied presence in the game environment (Van Looy et al. 2012), which has been shown to increase perceived enjoyment (Ahn and Noh 2024). Second, from the perspective of Self‐Discrepancy Theory (Higgins 1987), avatar identification may help reduce the gap between one's actual self and ideal self (İskender 2023; Klimmt et al. 2009), leading to more positive affect and elevated enjoyment.

Furthermore, a robust body of research has demonstrated that enjoyment is a strong positive predictor of consumption behavior in digital environments. In the context of gaming, perceived enjoyment not only directly increases players' willingness to purchase in‐game items but also indirectly does so by enhancing game loyalty (Hsiao and Chen 2016). Lehdonvirta (2009) identified hedonic value as one of the primary drivers of in‐game purchases. Taken together, these findings suggest that perceived enjoyment may represent an important experiential pathway linking avatar identification with OGC. Accordingly, the following hypothesis is proposed:

*Perceived enjoyment mediates the relationship between avatar identification and online game consumption*.

### 1.3 Avatar Identification and Avatar‐Related Consumption Values

If H2 is supported, the next question addressed by this study is: What consumption value perceptions associated with avatar identification may be linked to greater perceived enjoyment during gameplay? Drawing on the Theory of Consumption Values (Sheth et al. 1991), this study proposes that perceived consumption values associated with avatar enhancement may play an important role in this process. The Theory of Consumption Values conceptualizes consumer value as comprising functional value, social value, emotional value, epistemic value, and conditional value dimensions, which may influence consumer decision‐making (Cao et al. 2025). This framework has also been extended to virtual consumption. In online gaming, Park and Lee (2011) adapted the theory to include four dimensions of perceived value: visual authority value, enjoyment value, character competence value, and monetary value. Their findings suggest that higher perceptions of these values are positively associated with players' purchase intentions. Among these, enjoyment value and monetary value are more directly tied to general gameplay satisfaction and cost‐effectiveness of purchases, rather than to avatar identification per se (Park and Lee 2011). In contrast, visual authority value refers to the perceived benefits of customizing avatars, acquiring rare decorative items, or enhancing social visibility within the game community. Character competence value relates to the perceived benefits of improving an avatar's power, performance, or competitiveness (Park and Lee 2011). These two value dimensions are particularly relevant to avatar identification because they capture players' perceived benefits of expressing and enhancing aspects of the avatar that are personally meaningful. Thus, they provide a useful framework for examining how avatar identification may be associated with players' gaming experiences.

Avatars are central to self‐expression in online games, often reflecting players' emotional states, esthetic preferences, and ideal self‐image (Li et al. 2024). They also function as digital proxies for individuality and uniqueness (İskender 2023). From a self‐presentation perspective, players often design and customize their avatars to portray a “possible self” in virtual environments, seeking social recognition and elevated status through these portrayals (Lin and Wang 2014). When the avatar's visual attributes, such as style, appearance, or personality, align with an individual's ideal self, stronger emotional attachment and identification with the avatar are more likely to form (İskender 2023; Wang et al. 2024). Research has also shown that one of the main motivations for purchasing rare or decorative game items is to enhance personal appeal and distinguish oneself from others (Cai et al. 2022). Individuals with high levels of avatar identification tend to exhibit lower self‐concept clarity (Green et al. 2021), and those with lower self‐concept clarity are more prone to appearance‐based social comparison (Vartanian et al. 2025). Such individuals are more likely to seek self‐enhancement through external appearance adjustments. These findings suggest that players with stronger avatar identification may place greater value on visual attributes that facilitate self‐expression, self‐enhancement, and social visibility. Consequently, players with strong avatar identification may place greater value on visual authority, perceiving the acquisition of rare or visually striking items as a means of achieving their ideal self‐image. The successful acquisition of such items may enhance the player's visibility within the game environment and elicit admiration from others, thereby amplifying enjoyment of the gaming experience (Guo and Barnes 2009). Prior studies have shown that avatar customization contributes to perceived enjoyment, particularly through the visual satisfaction it provides (Turkay and Kinzer 2016). Thus, visual authority value may represent an important pathway through which avatar identification is associated with perceived enjoyment. Accordingly, the following hypothesis is proposed:

*Visual authority value mediates the relationship between avatar identification and perceived enjoyment*.

Players with a high level of avatar identification often perceive their avatars as extensions of the self within online environments (Van Looy et al. 2012). Online games are frequently characterized by competition and combat elements, in which avatar performance is closely tied to players' gaming experiences (Sun et al. 2024). For players who are strongly identified with their avatars, the avatar's performance may therefore carry greater personal significance. Empirical evidence indicates that avatar identification is positively associated with heightened expectations regarding the avatar's performance (Böffel et al. 2022), while players may purchase functional in‐game items to improve their avatars' performance and recover from negative gaming experiences (Cai et al. 2022). These findings suggest that players with stronger avatar identification may attach greater value to improving their avatars' competence and performance. When players believe that such purchases improve their avatar's competence, the resulting sense of achievement, control, and goal attainment contributes to greater enjoyment (Park and Lee 2011). From the perspective of Self‐Determination Theory (Deci and Ryan 1985), enhancing avatar competence satisfies the fundamental psychological need for competence, which in turn fosters intrinsic motivation and generates positive emotional outcomes, including increased perceived enjoyment. Accordingly, when players perceive a higher level of character competence value in their in‐game consumption, they may experience enhanced enjoyment and psychological satisfaction. On this basis, the following hypothesis is proposed:

*Character competence value mediates the relationship between avatar identification and perceived enjoyment*.

Building upon the preceding discussion, this study proposes that players with a high level of avatar identification are more likely to perceive OGC as a means of fulfilling both visual authority value and character competence value. These value perceptions may, in turn, be associated with greater perceived enjoyment during gameplay and, subsequently, with stronger consumption‐related responses. Prior research supports these proposed pathways, indicating that perceived value of in‐game items is positively associated with consumption behaviors in digital gaming environments (Guo and Barnes 2009; Hsiao and Chen 2016).

On this basis, the present study introduces a serial mediation model, in which visual authority value, character competence value, and perceived enjoyment serve as key mediators linking avatar identification to OGC. Specifically, we propose the following hypotheses:

*Character competence value and perceived enjoyment serially mediate the relationship between avatar identification and online game consumption*.

*Visual authority value and perceived enjoyment serially mediate the relationship between avatar identification and online game consumption*.

### 1.4 Overview

The hypothesized research model is presented in Figure 1. Two studies were conducted to test the model. Because the two studies used different research designs, OGC was assessed using different indicators. Study 1 used a questionnaire‐based approach and assessed participants' actual online game expenditure as the dependent variable. Study 2 focused on a specific character in *Honor of Kings* and assessed players' intention to purchase avatar‐related virtual items, providing further evidence for the proposed model. Together, the two studies examined the proposed associations using both an actual consumption measure and a consumption‐related behavioral intention.

**FIGURE 1:**
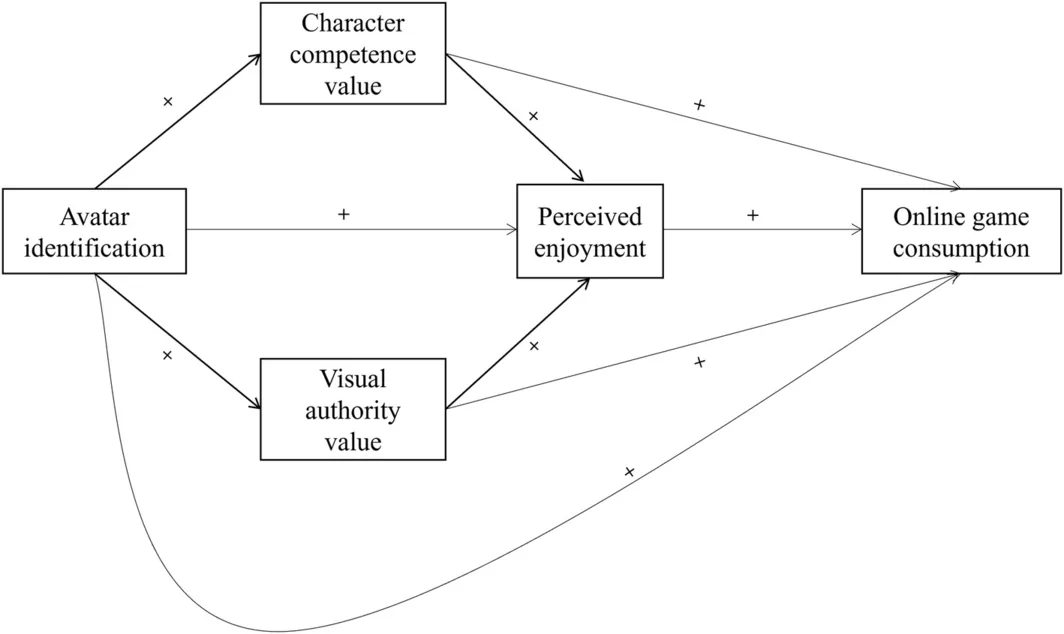
Hypothesis path diagram.

## 2 Study 1: Serial Mediation Model Testing in a General OGC Context

### 2.1 Participant

Participants were recruited through several university‐ and community‐based channels, as well as participant referrals. First, the researchers recruited participants through university courses by introducing the study and inviting eligible students to participate voluntarily. Second, the questionnaire was distributed through university online platforms to reach additional eligible participants. Third, participants were recruited from continuing education courses. Finally, participants who had completed the study could invite eligible friends who played online games to participate. Regardless of the recruitment source, all participants were required to meet the same eligibility criteria and completed the same questionnaire. Participation was voluntary, and informed consent was obtained before questionnaire completion. A total of 492 questionnaires were returned. After excluding 52 participants who reported not playing online games, 440 valid responses were retained, yielding an effective response rate of 89.4%. To evaluate the statistical sensitivity of the obtained sample, a sensitivity power analysis was subsequently conducted using G*Power. With a sample size of 440, *α* = 0.05, statistical power of 0.80, and seven predictors, the analysis indicated a minimum detectable effect size of 0.033. This suggests that the sample size provided adequate statistical sensitivity to detect relatively small effects.

The final sample consisted of individuals aged between 21 and 30 years (*M* = 23.1, SD = 1.1). The average monthly living expenditure of participants was CNY 1900.91 (approximately USD 264.66). Detailed demographic information and gaming characteristics about the participants is presented in Table 1. Before measuring avatar identification, we also asked participants to indicate their favorite game character. Participants' favorite game characters spanned a wide range of games, including Akali, Irelia, and Draven from *League of Legends*; Diaochan, Baili Shouyue, Xiao Qiao and Mai Shiranui from *Honor of Kings*; Gu Qinghan from *NARAKA: BLADEPOINT*; and Kamisato Ayaka from *Genshin Impact*, among others.

**TABLE 1:** Demographic and gaming characteristics of the participants (Study 1, *N* = 440).

Characteristics	Category	*n*	%
Gender	Male	285	64.8
	Female	155	35.2
Daily gaming duration	< 1 h	159	36.1
	1–3 h	216	49.1
	3–5 h	48	10.9
	> 5 h	17	3.9
Game type (multiple‐choice)	MOBA	346	78.6
	FPS	145	33.0
	ACT	9	2
	SLG	15	3.4
	Simulation and dress‐up games	18	4.1
	Otome games	31	7.0

### 2.2 Measures

#### 2.2.1 Avatar Identification

Avatar identification was measured using the 17‐item scale developed by Van Looy et al. (2012). Participants responded on a 5‐point Likert scale ranging from 1 (*strongly disagree*) to 5 (*strongly agree*), with higher scores indicating stronger identification with their in‐game avatar. A sample item is “My character is like me in many ways.” In this study, Cronbach's *α* was 0.95.

#### 2.2.2 Visual Authority Value

Visual authority value was measured using the 4‐item subscale from the OGC value scale developed by Park and Lee (2011). Participants rated each item on a 7‐point Likert scale ranging from 1 (*strongly disagree*) to 7 (*strongly agree*), with higher scores indicating greater perceived visual authority value. A simple item is “When I buy game items, I can adorn my game characters to be more fashionable or stylish.” In this study, Cronbach's *α* of the subscale was 0.82.

#### 2.2.3 Character Competence Value

Character competence value was measured using the three‐item subscale from the same consumption value scale developed by Park and Lee (2011). Participants responded on a 7‐point Likert scale ranging from 1 (*strongly disagree*) to 7 (*strongly agree*), with higher scores indicating greater perceived character competence value. A sample item is “When I buy game items, I can increase my game level quickly.” In this study, Cronbach's *α* of the subscale was 0.89.

#### 2.2.4 Perceived Enjoyment

Nabi and Krcmar (2004) proposed a tripartite model of media enjoyment, conceptualizing it as comprising cognitive, affective, and behavioral reactions. Based on this framework, the present study developed a self‐compiled Perceived Enjoyment scale to assess the extent to which players experience entertainment when using virtual items. The scale consists of 3 items rated on a 7‐point Likert scale from 1 (*strongly disagree*) to 7 (*strongly agree*), with higher scores indicating greater perceived enjoyment during gameplay. A sample item is: “When I use game items (e.g., equipment, skins, accessories), I experience more enjoyment.” In this study, Cronbach's *α* was 0.89.

#### 2.2.5 OGC

Participants' self‐reported total expenditure on online games over the past year, measured in Chinese Yuan (CNY), was used as an indicator of OGC.

### 2.3 Data Analysis

Descriptive statistics and Pearson's correlation analyses were first conducted for all major variables using SPSS 26.0 to examine the basic relationships among the constructs. Subsequently, confirmatory factor analysis (CFA) was performed on the latent variables within the measurement model using Amos 26.0 to assess convergent validity and determine whether the data met the assumptions required for structural equation modeling (SEM). After confirming satisfactory reliability and validity, the overall structural model was tested to evaluate model fit indices and the statistical significance of the hypothesized path coefficients. Finally, the PROCESS macro v4.1 in SPSS was employed to further examine mediation effects, clarifying the indirect pathways through which avatar identification was associated with OGC via proposed mediators.

### 2.4 Results

#### 2.4.1 Common Methods Bias

All data in this study were collected via self‐report measures, which may raise concerns regarding common method bias. To address this issue, Harman's single‐factor test was conducted. The results revealed five factors with eigenvalues greater than 1, and the first factor accounted for 38.41% of the total variance, which is below the recommended threshold of 50% (Podsakoff et al. 2003). This indicates that common method bias is unlikely to be a serious concern in the present study.

#### 2.4.2 Descriptive Statistics and Correlation Analysis

Descriptive statistics and correlation analysis were conducted, and the results are presented in Table 2. The average online game expenditure over the past year was CNY 372.3 (approximately USD 51.9), with a standard deviation of CNY 591.0 (approximately USD 82.8), indicating substantial variability in spending behavior across players. Moreover, avatar identification was found to be significantly and positively correlated with OGC, visual authority value, character competence value, and perceived enjoyment, supporting the suitability of further analyses to examine the hypothesized mediation pathways.

**TABLE 2:** Descriptive statistics and correlations analysis (Study 1, *N* = 440).

	*M* ± SD	1	2	3	4	5
1. Avatar identification	40.52 ± 16.42	—				
2. Visual authority value	18.44 ± 5.70	0.30***	—			
3. Character competence value	12.37 ± 5.20	0.27***	0.53***	—		
4. Perceived enjoyment	14.27 ± 4.76	0.26***	0.65***	0.56***	—	
5. Online game consumption (CNY)	372.3 ± 591.0	0.15**	0.14**	0.13**	0.26***	—

** *p* < 0.01.

*** *p* < 0.001.

#### 2.4.3 Validity Analysis

Prior to conducting SEM, the reliability and convergent validity of the measurement model were examined. Composite reliability (CR) was used to evaluate internal consistency, while average variance extracted (AVE) was employed to assess convergent validity. According to Fornell and Larcker (1981), *CR* values greater than 0.7 and *AVE* values greater than 0.5 indicate acceptable levels of reliability and convergent validity. All scales exhibited *CR* values above 0.7 and *AVE* values above 0.5, demonstrating satisfactory convergent validity and supporting the suitability of the measurement model for SEM analysis. The detailed results are presented in Table S1.

#### 2.4.4 Structural Equation Model Testing

SEM was conducted using Amos 26.0. The model demonstrated acceptable to good fit indices: *χ* ^*2*^/df = 3.630, *RMSEA* = 0.077, *CFI* = 0.911, *TLI* = 0.898, *IFI* = 0.911. Specifically, the *χ* ^2^/df was below the recommended threshold of 5, and *TLI* exceeded 0.8, indicating acceptable fit. Both *CFI* and *IFI* were above 0.9, and the *RMSEA* was below 0.08, suggesting a good overall model fit. Therefore, the proposed structural model was deemed to adequately fit the observed data. The standardized path coefficients for all hypothesized relationships are presented in Figure 2.

**FIGURE 2:**
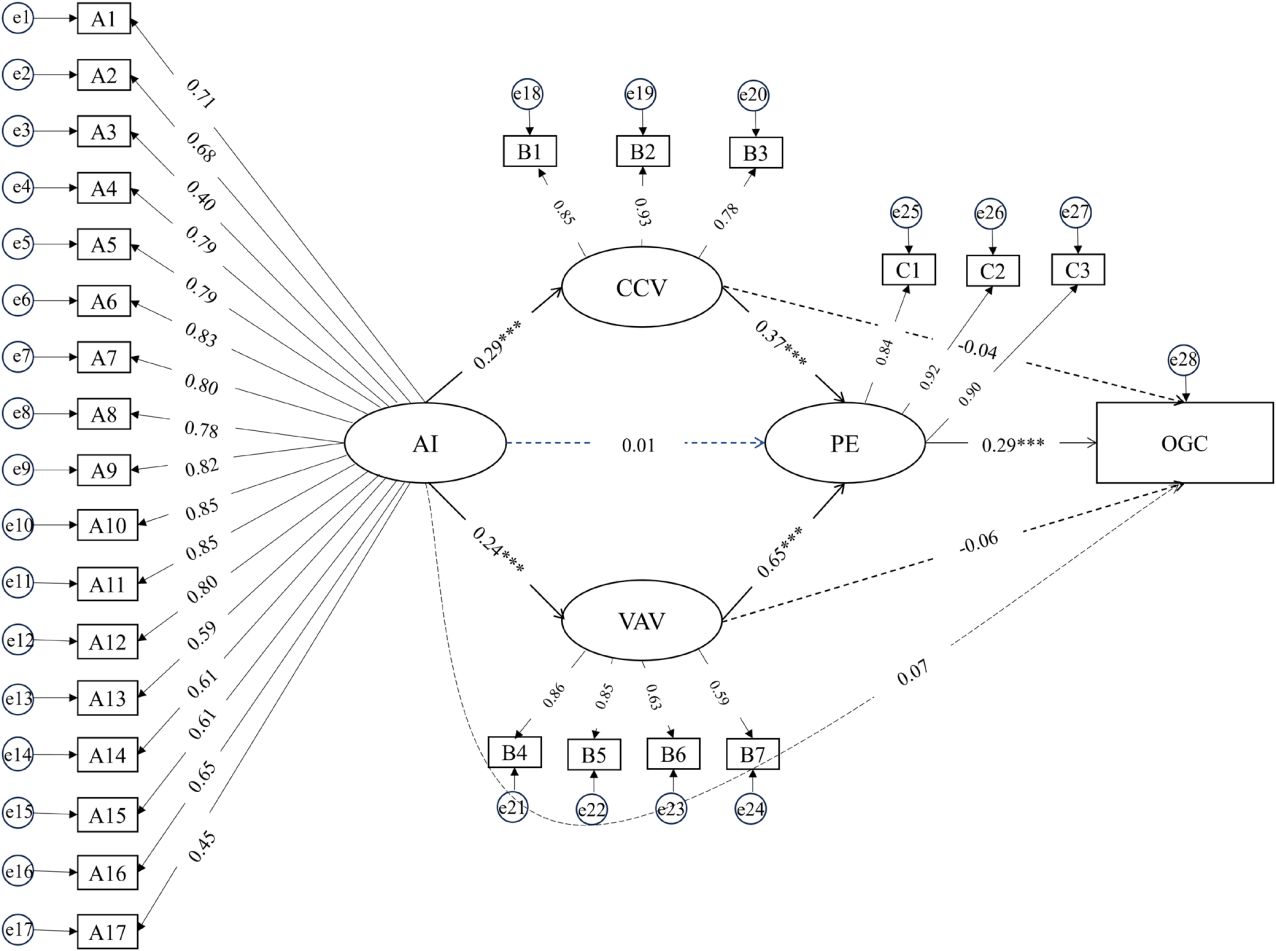
Standardized path coefficient diagram of the model (Study1). AI, avatar identification; CCV, character competence value; OGC, online game consumption; PE, perceived enjoyment; VAV, visual authority value.

Given the positively skewed distribution of OGC, characterized by a substantial proportion of zero‐consumption observations and a small number of relatively high‐consumption observations, we conducted an additional robustness analysis. Specifically, OGC was logarithmic transformed using ln(OGC + 1), which accommodates zero‐consumption observations while reducing the influence of high‐consumption observations. The proposed SEM was then re‐estimated using the transformed measure as the outcome variable. The results were highly consistent with those obtained using the original OGC measure, with the hypothesized structural paths and indirect effects showing the same pattern of significance and substantive interpretation. These findings suggest that the observed relationships were not substantially driven by the skewed distribution of OGC.

#### 2.4.5 Test of the Mediation Model

To further validate the proposed model, mediation analysis was conducted using Model 80 in the PROCESS macro v4.1 for SPSS. Avatar identification was specified as the independent variable; visual authority value, character competence value, and perceived enjoyment were entered as mediators; and OGC served as the dependent variable. Gender, age, and monthly living expenditure were included as control variables. The results are presented in Table 3.

**TABLE 3:** Standardized Regression Coefficients for the Mediation Model (Study1, *N* = 440).

	VAV			CCV			PE			OGC		
	(Mediating Variable 1)			(Mediating Variable 2)			(Mediating Variable 3)			(dependent variable)		
	*β*	*t*	95% CI	*β*	*t*	95% CI	*β*	*t*	95% CI	*β*	*t*	95% CI
Gender	−0.10	−0.93	[−1.59, 0.57]	−0.06	−1.25	[−1.61, 0.36]	−0.04	−1.04	[−1.03, 0.32]	−0.21	−4.65***	[−371, −151]
Age	−0.04	0.39	[−0.38.0.57]	−0.07	−2.71**	[−0.79, 0.08]	0.02	0.69	[−0.19, 0.40]	0.02	0.46	[−37.2, 60.0]
MLE	0.05	0.28	[−0.01, 0.01]	0.05	1.13	[−0.01, 0.01]	0.08	2.25*	[0.00, 0.01]	0.09	2.04*	[0.002, 0.10]
AI	0.29	6.38***	[0.07, 0.13]	0.27	5.85***	[0.06, 0.11]	0.03	0.87	[−0.01, 0.03]	0.10 (0.14)	2.09* (3.03**)	[0.21, 6.94] [1.76, 8.52]
VAV							0.49	11.78***	[0.34, 0.47]	−0.06	−1.00	[−19.0, 6.32]
CCV							0.29	6.97***	[0.19, 0.34]	−0.02	−0.43	[−15.5, 9.96]
PE										0.26	4.07***	[16.5, 47.3]
*R* ^2^	0.09			0.09			0.49			0.13		
*F*	10.67***			10.10***			70.45***			9.16***		

*Note:* The coefficient in parentheses represents the regression coefficient of AI on OGC when the mediating variables were not included in the model.

Abbreviations: AI, avatar identification; CCV, character competence value; MLE, monthly living expenditure; OGC, online game consumption; PE, perceived enjoyment; VAV, visual authority value.

* *p* < 0.05.

** *p* < 0.01.

*** *p* < 0.001.

In the regression model excluding mediators, avatar identification had a significant positive association with OGC (*β* = 0.14, *t* = 3.03, 95% CI [1.76, 8.52]), supporting H1. After the inclusion of mediating variables, avatar identification significantly and positively predicted both visual authority value (*β* = 0.29, *t* = 6.38, 95% CI [0.07, 0.13]) and character competence value (*β* = 0.27, *t* = 5.85, 95% CI [0.06, 0.11]). In turn, both visual authority value (*β* = 0.49, *t* = 11.78, 95% CI [0.34, 0.47]) and character competence value (*β* = 0.29, *t* = 6.97, 95% CI [0.19, 0.34]) significantly predicted perceived enjoyment. Perceived enjoyment then significantly predicted OGC (*β* = 0.26, *t* = 4.07, 95% CI [16.5, 47.3]). The direct effect of avatar identification on perceived enjoyment was not significant (*p* = 0.38). Similarly, neither visual authority value (*p* = 0.33) nor character competence value (*p* = 0.67) directly predicted OGC, suggesting that their associations with consumption were fully mediated by perceived enjoyment.

The total effect of avatar identification on OGC was 5.01, the direct effect was 3.58, and the indirect effect was 1.43. The indirect effect accounted for 28.5% of the total effect. Among the five indirect paths examined, two were statistically significant, with 95% confidence intervals that did not include 0. Specifically: Ind4 (avatar identification → visual authority value → perceived enjoyment → OGC) yielded an indirect effect of 1.31, 95% CI [0.70, 2.13]; Ind5 (avatar identification → character competence value → perceived enjoyment → OGC) yielded an indirect effect of 0.71, 95% CI [0.34, 1.22]. These findings support both H5a and H5b. In contrast, Ind1(avatar identification → perceived enjoyment → OGC) (effect = 0.29, 95% CI [−0.38, 2.13]), Ind2 (avatar identification → visual authority value → OGC) (effect = −0.64, 95% CI [−2.06, 0.68]), and Ind3 (avatar identification → character competence value → OGC) (effect = −0.23, 95% CI [−1.43, 0.83]) were not statistically significant, as their 95% confidence intervals included 0.

These findings indicate that avatar identification was not directly associated with OGC in the mediation model and was not indirectly associated with consumption through value perceptions alone. Instead, the findings support the proposed serial mediation pathways involving visual authority value or character competence value, followed by perceived enjoyment. This pattern highlights the potential role of perceived enjoyment in linking avatar identification to consumption behavior and provides support for the serial mediation mechanism proposed in the theoretical model.

## 3 Study 2: Serial Mediation Model Testing in a Specific Online Game

Study 2 aimed to further test the relationships among the key variables in the proposed model by focusing on a specific game character. The study was conducted in the context of *Honor of Kings*, one of the most popular online games in China, which reports approximately 140 million monthly active users (Liu et al. 2023). The selected avatar was Sun Shangxiang, one of the game's most frequently used heroes, with a pick rate of 21.3%, ranking among the top across all available avatars. Participants with prior gameplay experience in *Honor of Kings* were recruited and were instructed to respond to questionnaire items based on their actual experiences and perceptions related to this character. By focusing on a well‐known character within a real‐world gaming environment, Study 2 provided a more ecologically grounded context for further examining the robustness of the proposed model and the relationship between avatar identification and OGC.

### 3.1 Participant

Data for Study 2 were collected through the online survey platform Wenjuanxing (www.wjx.com). A total of 251 responses were received. After excluding participants who reported no experience playing *Honor of Kings* and those whose response times were excessively long (> 600 s) or extremely short (< 200 s), 229 valid responses were retained, yielding an effective response rate of 87.7%. To evaluate the statistical sensitivity of the obtained sample, a sensitivity power analysis was subsequently conducted using G*Power. With a sample size of 229, *α* = 0.05, statistical power of 0.80, and seven predictors, the analysis indicated a minimum detectable effect size of 0.067. This suggests that the sample size provided adequate statistical sensitivity to detect relatively small effects.

Among the valid participants, 119 were male and 110 were female, with a mean age of 29.8 years (SD = 8.0). The average monthly living expenditure was CNY 2730.79 (approximately USD 380.31). Participants reported an average cumulative in‐game expenditure of CNY 3398.98 (approximately USD 473.08) on *Honor of Kings*. Regarding daily gameplay duration, 45 participants (19.7%) played less than 1 h per day, 142 participants (62.0%) played 1–3 h, 38 participants (16.6%) played 3–5 h, and 4 participants (1.7%) reported playing more than 5 h per day.

### 3.2 Procedure and Measurements

Participants were first presented with an image of the Sun Shangxiang from Honor of Kings, with the character's name clearly labeled. They were then instructed to imagine themselves playing the game using this specific character. Subsequently, participants completed a set of questionnaires pertaining to this character, including measures of avatar identification, perceived visual authority value, perceived character competence value, and perceived enjoyment when using the avatar. The measurement instruments were consistent with those employed in Study 1; however, the item wordings were adapted to fit the context of the specific game and character. For example, items were rephrased as “Sun Shangxiang is like me in many ways.” Cronbach's *α*'s for each scale in this study were as follows: avatar identification (*α* = 0.95), visual authority value (*α* = 0.65), character competence value (*α* = 0.80), and perceived enjoyment (*α* = 0.76). Although the internal consistency of the visual authority value measure was slightly below the conventional threshold of 0.70, it was adapted from an established and widely used scale. Given its established theoretical and empirical basis, we retained the measure while acknowledging its relatively low reliability as a limitation.

To assess OGC intention, a scenario‐based simulation task was employed. Given the scenario‐based experimental design, in‐game purchase intention was assessed rather than actual expenditure. Specifically, participants' self‐reported likelihood of purchasing the avatar‐related virtual items presented in the experimental scenario was used as an indicator of OGC. Although purchase intention is conceptually distinct from actual expenditure, it provides a proximal indicator of consumers' behavioral tendency in response to specific purchase stimuli. Participants were presented with the following prompt: “You are browsing the in‐game store in *Honor of Kings* and come across various purchasable items related to Sun Shangxiang (e.g., skins). You are free to choose whether to purchase these items. You will now see several products (see Figure 3). Please rate how likely you are to purchase each item.” Participants rated their purchase intention for five real in‐game items, each displayed with images and prices (both virtual currency and real‐money equivalents). Purchase intention for each item was measured on a 5‐point Likert scale ranging from 1 (*very unlikely*) to 5 (*very likely*), with higher scores indicated stronger purchase intention. The internal consistency for this 5‐item purchase intention scale was 0.75. A composite score was computed and used as the outcome variable representing in‐game purchase intention.

**FIGURE 3:**
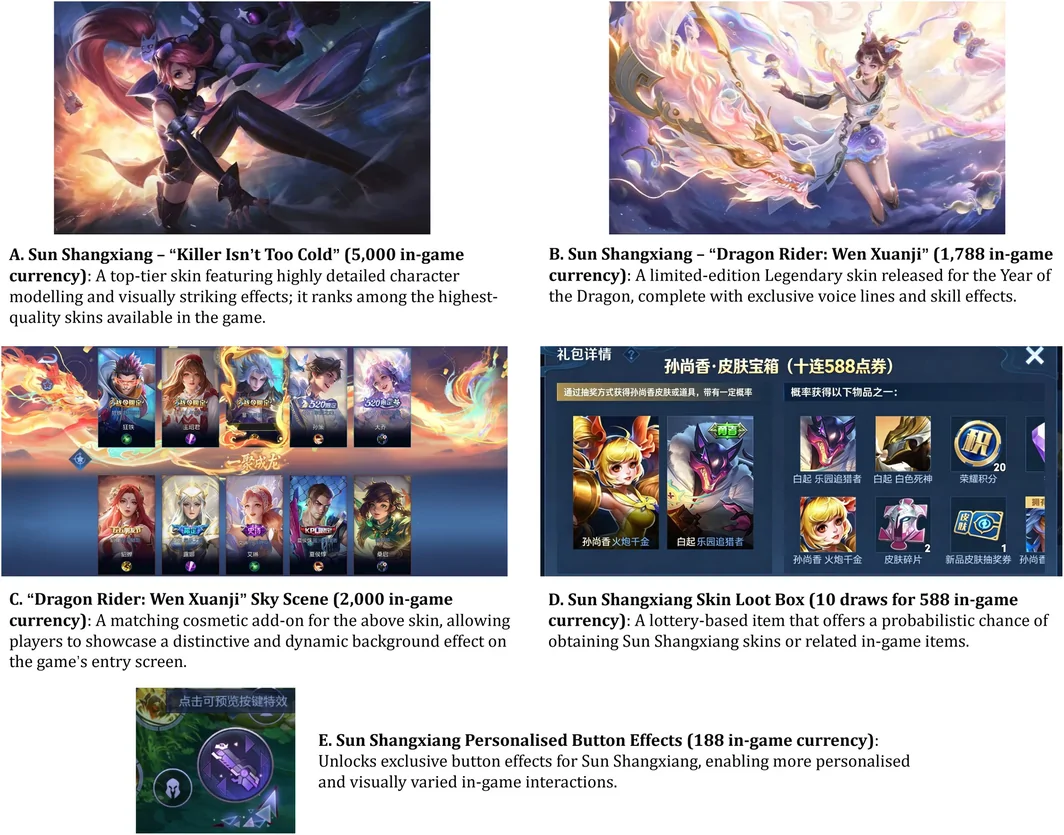
Available items for Sun Shangxiang in *Honor of Kings* (10 in‐game currency = 1CNY).

At the end of the survey, participants provided demographic information and reported their cumulative past expenditure in *Honor of Kings*.

### 3.3 Results

#### 3.3.1 Common Methods Bias

To assess the potential impact of common method bias, Harman's single‐factor test was conducted. The results revealed that five factors had eigenvalues greater than 1, and the largest single factor accounted for 39.62% of the total variance, which is below the 50% threshold recommended by Podsakoff et al. (2003). These findings suggest that common method bias is not a serious concern in this study.

#### 3.3.2 Descriptive Statistics and Correlation Analysis

Descriptive statistics and correlation analysis were conducted for all variables, and the results are presented in Table 4. All variables were significantly correlated (*p* < 0.001), consistent with the findings of Study 1, thus supporting the feasibility of subsequent model testing.

**TABLE 4:** Descriptive statistics and correlations analysis (Study 2, *N* = 229).

	*M* ± SD	1	2	3	4	5
1. Avatar identification	63.72 ± 14.11	—				
2. Visual authority value	23.44 ± 2.77	0.34***	—			
3. Character competence value	16.77 ± 2.98	0.53***	0.40***	—		
4. Perceived enjoyment	17.56 ± 2.52	0.56***	0.55***	0.54***	—	
5. Online game consumption (intention)	19.21 ± 3.72	0.55***	0.40***	0.47***	0.55***	—

*** *p* < 0.001.

#### 3.3.3 Mediation Analysis

A mediation analysis was conducted using Model 80 of PROCESS v4.1. Avatar identification was entered as the independent variable; visual authority value, character competence value, and perceived enjoyment were entered as mediators; and OGC intention served as the dependent variable. Gender, age, monthly living expenditure, and cumulative expenditure in *Honor of Kings* were included as control variables. The results are presented in Table 5.

**TABLE 5:** Standardized regression coefficients for the mediation model (Study 2, *N* = 229).

	VAV			CCV			PE			OGC		
	(Mediating Variable 1)			(Mediating Variable 2)			(Mediating Variable 3)			(dependent variable)		
	*β*	*t*	95% CI	*β*	*t*	95% CI	*β*	*t*	95% CI	*β*	*t*	95% CI
Gender	−0.10	−1.54	[−0.44, 0.05]	−0.06	−1.09	[−0.34, 0.10]	0.01	0.21	[−0.17, 0.21]	0.03	0.55	[−0.15, 0.26]
Age	−0.04	−0.65	[−0.02, 0.01]	−0.02	−0.29	[−0.02, 0.01]	−0.05	−1.09	[−0.02, 0.01]	−0.002	−0.05	[−0.01, 0.01]
MLE	0.04	0.59	[−0.001, 0.001]	−0.08	−1.27	[−0.01, 0.00]	−0.03	−0.63	[0.00, 0.00]	−0.02	−0.53	[−0.001, 0.01]
CI	0.10	1.41	[0.000, 0.001]	0.09	1.49	[0.00, 0.00]	0.04	0.77	[0.00, 0.00]	0.08	1.47	[0.00, 0.00]
AI	0.34	5.33***	[0.21, 0.47]	0.54	9.26***	[0.42, 0.65]	0.33	5.69***	[0.21, 0.45]	0.31 (0.55)	4.49*** (9.61***)	[0.17, 0.44] [0.44, 0.66]
VAV							0.35	6.55***	[0.24, 0.45]	0.09	1.49	[−0.01, 0.21]
CCV							0.22	3.77***	[0.11, 0.34]	0.12	1.94	[−0.03, 0.22]
PE										0.25	3.42***	[0.11, 0.39]
*R* ^2^	0.14			0.30			0.50			0.42		
*F*	7.21***			18.80***			31.69***			19.67***		

*Note:* The coefficient in parentheses represents the regression coefficient of AI on OGC when the mediating variables were not included in the model.

Abbreviations: AI, avatar identification; CCV, character competence value; CI, consumption in *Honor of Kings*; MLE, monthly living expenditure; PE, perceived enjoyment; VAV, visual authority value.

*** *p* < 0.001.

In the regression model excluding mediating variables, avatar identification was significantly and positively associated with OGC (*β* = 0.55, *t* = 9.61, 95% CI [0.44, 0.66]). After introducing the mediators, avatar identification remained a significant positive predictor of OGC (*β* = 0.31, *t* = 4.49, 95% CI [0.17, 0.44]). Avatar identification also significantly predicted both visual authority value (*β* = 0.34, *t* = 5.33, 95% CI [0.21, 0.47]) and character competence value (*β* = 0.54, *t* = 9.26, 95% CI [0.42, 0.65]). Both visual authority value (*β* = 0.35, *t* = 6.55, 95% CI [0.24, 0.45]) and character competence value (*β* = 0.22, *t* = 3.77, 95% CI [0.11, 0.34]) significantly predicted perceived enjoyment, which in turn significantly predicted OGC (*β* = 0.25, *t* = 3.42, 95% CI [0.11, 0.39]). However, neither visual authority value (*p* = 0.17) nor character competence value (*p* = 0.10) directly predicted OGC, indicating that their associations with consumption were accounted for by perceived enjoyment. These results are basically consistent with the findings of Study 1. However, Study 2 additionally revealed a significant direct association between avatar identification and perceived enjoyment (*β* = 0.33, *t* = 5.69, 95% CI [0.21, 0.45]). The standardized regression path coefficients are illustrated in Figure 4.

**FIGURE 4:**
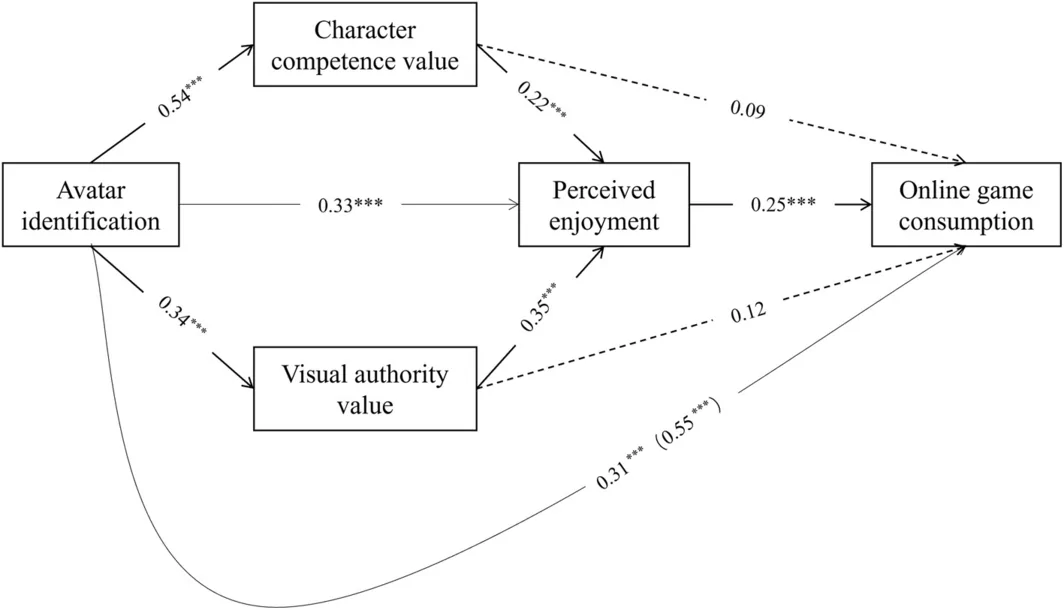
Standardized path coefficient diagram of the model (Study2).

The total effect was 0.55, the direct effect was 0.31, and the indirect effect was 0.24, all statistically significant. The indirect effect accounted for 43.6% of the total effect. Among the five tested paths, Ind1 (avatar identification → perceived enjoyment → OGC) (effect = 0.08, 95% CI [0.01, 0.16]) was significant, indicating that avatar identification was indirectly associated with consumption through perceived enjoyment, thus supporting H2. Ind4 (effect = 0.03, 95% CI [0.01, 0.06]) and Ind5 (effect = 0.03, 95% CI [0.01,0.06]) were also significant, with confidence intervals not including 0, supporting the serial mediation effects and providing support for H5a and H5b. In contrast, Ind2 (effect = 0.03, 95% CI [−0.02, 0.09]) and Ind3 (effect = 0.03, 95% CI [−0.03, 0.18]) were not significant, consistent with the results of Study 1, suggesting that both visual authority value and character competence value were not directly associated with OGC, but showed indirect associations through perceived enjoyment.

## 4 Discussion

### 4.1 Main Finding of the Study

This study, conducted through two sub‐studies, investigated the relationship between avatar identification and OGC, focusing on the value‐driven and psychological experience pathways. Study 1, employing a questionnaire‐based approach, revealed a serial mediation model in which avatar identification was associated with visual authority value and character competence value, which were in turn associated with perceived enjoyment and, subsequently, OGC. Study 2 further tested this model by selecting a specific character from a specific game to enhance the robustness and external validity of the findings.

The results indicate that avatar identification significantly and positively predicted OGC, suggesting that individuals with higher levels of avatar identification tend to report greater online game spending. This finding aligns with previous research (Sharma 2024). Mediation analysis further revealed that avatar identification was indirectly associated with OGC through perceived enjoyment. These results extend existing literature by providing insight into the psychological processes linking avatar identification with consumption behavior in online games. Specifically, individuals with high levels of avatar identification may experience stronger emotional connections with their avatars and report greater enjoyment during gameplay (Klimmt et al. 2009). Such enjoyment may represent an important experiential correlate of OGC (Lehdonvirta 2009). Building on the Theory of Consumption Values, further analysis identified two key mediators linking avatar identification to perceived enjoyment: visual authority value and character competence value. This finding reveals two potentially important value‐related aspects of OGC: self‐presentation and competence. As the sole representative of the player in the virtual world, an avatar's appearance and behavior embody the individual's pursuit of an ideal self (Dunn and Guadagno 2012). Purchasing character cosmetics, rare skins, and other visually dominant elements may provide opportunities for self‐presentation and status expression, which may be associated with more enjoyable gaming experiences. Similarly, performance‐enhancing items may provide players with opportunities to experience competence and achievement within the game. High levels of avatar identification foster a stronger emotional bond with the avatar, leading to shared emotional experiences: players feel joy and frustration in tandem with their avatar's outcomes (Van Looy et al. 2012; Wang et al. 2024). As avatar abilities improve through in‐game purchases, players are more likely to succeed in challenging tasks and earn rewards, which, in turn, heightens their perceived enjoyment of the game (Uysal and Yildirim 2016). These findings suggest that avatar‐related value perceptions may be relevant to players' enjoyment of the gaming experience.

Moreover, this study empirically validated two serial mediation pathways: (1) avatar identification → visual authority value → perceived enjoyment → OGC, and (2) avatar identification → character competence value → perceived enjoyment → OGC. Specifically, individuals with high levels of avatar identification tended to report stronger value perceptions related to their avatars' status, attractiveness, or functional capabilities, which were associated with greater perceived enjoyment and, in turn, stronger consumption‐related responses. These findings suggest that perceived enjoyment may represent an important experiential link between avatar‐related consumption values and OGC.

Although the two studies generally supported the proposed model, some differences emerged in the specific pathways. In the mediation model of Study 1, avatar identification was not directly associated with either perceived enjoyment or OGC; instead, its associations with these outcomes were primarily transmitted through perceived consumption values. In the mediation model of Study 2, by contrast, avatar identification was directly associated with both perceived enjoyment and purchase intention. These differences may partly reflect the different contexts and outcome measures used across the two studies. One possible explanation concerns the different avatars examined in the two studies. In Study 1, participants evaluated their favorite game characters, with whom they were likely to have developed familiarity, preference, and emotional attachment through their prior gaming experiences (Banks and Bowman 2021). Their responses may therefore have been shaped by a broader range of prior gaming experiences and motives for interacting with and enhancing their avatars (Cleghorn and Griffiths 2015). In this context, avatar identification may not have been sufficient to directly predict enjoyment or consumption after accounting for the perceived consumption values. Instead, identification may first increase the perceived importance of the avatar's appearance or competence, which may subsequently contribute to enjoyment and consumption. In Study 2, however, all participants evaluated the same assigned game character, which provided a more standardized and controlled context. This may have reduced variation arising from differences in participants' previous experiences with those characters. As a result, identification with the target character may have represented a more immediate psychological connection within the study context, making its association with enjoyment more direct. Moreover, Study 2 assessed purchase intention rather than actual online game expenditure. Purchase intention may represent a more proximal indicator of behavioral motivation than actual expenditure, as behavioral intentions are generally regarded as an immediate antecedent of actual behavior (Ajzen 1991). In the context of online games, previous research has also shown that avatar identification can be associated with players' intentions to purchase virtual items (Wu and Hsu 2018). Thus, compared with actual expenditure, purchase intention may be more immediately responsive to players' psychological experiences associated with their avatars, which may partly explain why avatar identification showed a more direct association with purchase intention in Study 2.

### 4.2 Theoretical Implications

First, this study broadens the psychological perspective on OGC. While prior research has largely focused on factors such as service quality (Yang et al. 2009), player loyalty (Teng 2018), and motivations related to escapism or emotional involvement (Hussain et al. 2024), there has been limited attention to the role of game avatars in players' self‐concept and identity‐related consumption. Avatar identification has traditionally been examined in relation to psychological outcomes such as internet gaming disorder (Yang et al. 2025) and self‐concept clarity (Green et al. 2021), whereas its association with consumer decision‐making in online gaming remains underexplored. This study extends research on virtual identity by showing that players' relationships with their avatars are also relevant to consumption‐related responses, providing a novel perspective on virtual consumption.

Second, this study extends the application boundaries of self‐related theories within the context of online gaming. The concept of virtual identity aligns with Extended‐Self Theory (Belk 2013), wherein avatars function not only as projections of the self but also as objects through which individuals express aspects of identity and pursue personally meaningful values. Likewise, Self‐Determination Theory (Deci and Ryan 1985) provides a valuable framework for understanding why virtual environments may make certain consumption experiences psychologically meaningful. Online games offer high levels of autonomy, personalization, and goal‐oriented activities, supporting players' needs for autonomy and competence (Tyack and Mekler 2024). Purchasing virtual equipment and cosmetic items may provide opportunities for self‐expression and demonstrating competence, suggesting that virtual consumption may be understood as part of players' self‐related experiences in digital environments.

Third, the findings further suggest that consumption value may be connected with spending behavior through an experiential process involving perceived enjoyment. The findings indicate that perceived enjoyment may represent an important experiential component in the association between avatar‐related consumption values and consumption‐related outcomes. Previous research has established perceived consumption value as an important determinant of players' willingness to purchase virtual items (Park and Lee 2011). The present findings extend this perspective by suggesting a value–experience–consumption process, in which avatar‐related consumption values are associated with more enjoyable gaming experiences and stronger consumption‐related responses.

### 4.3 Practical Implications

The findings offer practical implications for online game design by highlighting the potential relevance of avatar identification and perceived consumption values to players' enjoyment and consumption‐related responses. When in‐game items fulfill players' visual authority value or character competence value, they may be perceived as more attractive and may be more likely to stimulate purchase intentions. These findings suggest that game designers may consider how the visual and functional attributes of in‐game items correspond to players' identity‐related and value‐based preferences.

For example, designers could consider integrating distinctive visual features with gameplay achievements, such as linking rare skins with the completion of high‐level challenges. Similarly, equipment with distinctive visual effects could be used to communicate strength or expertise. Such design approaches may provide players with opportunities to express both esthetic preferences and perceived competence through their avatars. However, these possibilities should be viewed as design considerations rather than established strategies for increasing spending, particularly given the modest amount of variance in spending behavior explained by the model.

## 5 Limitation and Suggestion for Future Research

First, to examine two self‐enhancement related values: visual authority value and character competence value, both of which have been identified in prior research as key drivers of enjoyment and consumption in online games (Hamari and Keronen 2017; Park and Lee 2011). However, other scholars have proposed additional value dimensions from different perspectives. For instance, Mosavi (2012) introduced convenience value, defined as the immediate and effortless satisfaction of consumer needs, and epistemic value, which refers to curiosity, novelty, or knowledge acquisition, both of which significantly influence online purchasing behavior. Similarly, Kim et al. (2011) identified the roles of functional, emotional, and social values in shaping consumer decisions. While these dimensions may not be directly linked to self‐concept or self‐enhancement, they remain closely associated with consumers' online purchasing tendencies. Future research could further explore how these diverse value dimensions interact and jointly influence decision‐making in OGC, thereby contributing to a more comprehensive and integrated theoretical model of virtual consumer behavior.

Second, the formation and functional mechanisms of avatar identification are inherently complex. It may stem from the projection of the ideal self onto the virtual self (Bessière et al. 2007), or develop through growing emotional and cognitive bonds with media characters (Van Looy et al. 2012). Previous studies have examined avatar identification both as an antecedent of Internet gaming disorder (Zhang et al. 2024) and as a moderator of the relationship between depression and gaming disorder (Burleigh et al. 2018). In the present study, avatar identification was treated as an independent variable predicting perceived value, enjoyment, and avatar‐related consumption. However, the psychological mechanisms underlying these associations may be more complex than those examined here. For example, avatar identification may be associated with the representation and pursuit of the ideal self (Li et al. 2024; Mancini et al. 2019), potentially making avatar‐related purchases more relevant to self‐expression and ideal‐self enhancement. These theoretical constructs, however, were not directly assessed in the present study. Future research should directly examine these potential mechanisms and the possible moderating role of avatar identification.

Finally, several methodological limitations should be acknowledged. First, this study employed a cross‐sectional design, limiting its ability to capture causal or temporal dynamics among the variables. Future research could adopt longitudinal designs or experimental manipulations to more precisely identify causal mechanisms. Second, the sample was relatively homogeneous in age, which may affect the generalizability of the findings. Although young adults are a core demographic for online gaming (Chen 2010), consumption patterns often differ significantly across age groups (Duffett and Mxunyelwa 2025; Zhou et al. 2007). Future studies should seek more diverse samples across age groups to enhance representativeness. Additionally, cultural background may influence how individuals perceive and evaluate virtual goods (Hofstede 2001). In different sociocultural contexts, players may ascribe different meanings to visual and competence values, resulting in varying consumption behaviors. Cross‐cultural research could therefore examine the applicability of the proposed model across regions to enhance its external validity. Thirdly, although Study 2 used the same well‐known game character for all participants, participants may have differed in their pre‐existing familiarity with and liking of the character, which could have contributed to the observed associations. Future research should directly assess and statistically control for these factors when using a standardized‐character paradigm. Finally, another limitation concerns the relatively low internal consistency of the visual authority value measure in Study 2 (*α* = 0.65). Although the measure was adapted from a previously established scale and demonstrated satisfactory reliability in Study 1 (*α* = 0.82), its lower reliability in Study 2 may have introduced additional measurement error and attenuated the estimated associations involving visual authority value. Future research should further validate the adapted measure and examine whether the observed associations can be replicated using more reliable measures.

## 6 Conclusion

This study proposed and tested a serial mediation model to examine the associations between avatar identification and OGC. Results show that players with stronger avatar identification were more likely to engage in self‐expressive or performance‐based purchases to enhance enjoyment, which in turn were associated with greater spending. Two complementary studies provided convergent evidence for the proposed model and supported its robustness across different research contexts. These findings shed new light on the psychological processes linking virtual identity with virtual consumption, offering implications for both theory and practice. As metaverse platforms continue to expand, avatars are no longer confined to a single game but serve as cross‐platform extensions of the self, playing increasingly central roles in self‐expression, social interaction, and virtual consumption. Understanding the psychological processes linking avatar identification with value‐based consumption not only deepens our theoretical understanding of virtual consumer psychology but also informs the strategic development of more engaging, personalized, and commercially viable digital environments.

## Funding

The authors have nothing to report.

## Ethics Statement

All procedures performed in studies involving human participants were in accordance with the ethical standards of the institutional and/or national research committee and with the 1964 Helsinki Declaration and its later amendments or comparable ethical standards. Ethical approval was obtained on December 24, 2024, from the Psychological Ethics Committee of China University of Geosciences (Beijing) [Grant No. 2024122401].

## Consent

All participants were informed of the purpose of the study, the voluntary nature of their participation, and that their data would only be used for academic research and will be published in anonymized form. Consent was obtained by checking a consent box. The survey was anonymous, and no personally identifiable information was collected. All participants were competent adults; no minors or vulnerable individuals were involved. Informed consent was obtained from all individual participants included in the study.

## Conflicts of Interest

The authors declare no conflicts of interest.

## Supporting information


**Table S1:** Composite reliability and convergent validity indices (*N* = 440).

## Data Availability

The data that support the findings of this study are available from the corresponding author upon reasonable request.
