# Supplementary material for: Playing the Self, Paying the Price: A Dual‐Path Model of Avatar Identification and Online Game Consumption
Source: Psych J. 2026 Oct 15;15(5):e70125. doi: 10.1002/pchj.70125 (PMC13671006; doi:10.1002/pchj.70125)
Supplement: Supplementary file 1 [file PCHJ-15-e70125-s001.docx]

**Table S1.** Composite Reliability and Convergent Validity Indices (*N*=440)

| **Variables** | **Item** | **Significance Estimation** | | | |  | |  |  |
| --- | --- | --- | --- | --- | --- | --- | --- | --- | --- |
|  |  |  |  |  |  |  |  |  |  |
|  |  | ***UnStd*** | ***S.E.*** | ***z-value*** | ***p*** | ***Std*** | ***SMC*** | ***CR*** | ***AVE*** |
|  |  |  |  |  |  |  |  |  |  |
| AI | A1 | 1.10 | 0.83 | 13.22 | *** | 0.71 | 0.50 | 0.95 | 0.52 |
|  | A2 | 1.07 | 0.08 | 12.83 | *** | 0.68 | 0.46 |  |  |
|  | A3 | 0.64 | 0.08 | 7.93 | *** | 0.40 | 0.16 |  |  |
|  | A4 | 1.23 | 0.09 | 14.42 | *** | 0.79 | 0.62 |  |  |
|  | A5 | 1.21 | 0.08 | 14.46 | *** | 0.79 | 0.62 |  |  |
|  | A6 | 1.35 | 0.09 | 14.61 | *** | 0.80 | 0.64 |  |  |
|  | A7 | 1.32 | 0.09 | 14.39 | *** | 0.78 | 0.61 |  |  |
|  | A8 | 1.20 | 0.08 | 15.04 | *** | 0.83 | 0.69 |  |  |
|  | A9 | 1.32 | 0.09 | 14.85 | *** | 0.82 | 0.66 |  |  |
|  | A10 | 1.35 | 0.09 | 15.37 | *** | 0.85 | 0.73 |  |  |
|  | A11 | 1.24 | 0.08 | 15.39 | *** | 0.85 | 0.73 |  |  |
|  | A12 | 1.27 | 0.09 | 15.64 | *** | 0.80 | 0.64 |  |  |
|  | A13 | 0.90 | 0.08 | 11.35 | *** | 0.59 | 0.35 |  |  |
|  | A14 | 0.96 | 0.08 | 11.64 | *** | 0.61 | 0.37 |  |  |
|  | A15 | 1.01 | 0.09 | 11.74 | *** | 0.62 | 0.38 |  |  |
|  | A16 | 1.00 |  |  |  | 0.65 | 0.42 |  |  |
|  | A17 | 0.83 | 0.09 | 8.90 | *** | 0.45 | 0.20 |  |  |
| PGE | B1 | 0.96 | 0.04 | 26.10 | *** | 0.86 | 0.73 | 0.93 | 0.81 |
|  | B2 | 1.06 | 0.03 | 31.29 | *** | 0.93 | 0.86 |  |  |
|  | B3 | 1.00 |  |  |  | 0.91 | 0.83 |  |  |
| VAV | B4 | 1.13 | 0.08 | 14.57 | *** | 0.85 | 0.72 | 0.82 | 0.53 |
|  | B5 | 1.06 | 0.07 | 14.37 | *** | 0.83 | 0.69 |  |  |
|  | B6 | 1.00 |  |  |  | 0.66 | 0.43 |  |  |
|  | B7 | 0.83 | 0.08 | 10.11 | *** | 0.54 | 0.29 |  |  |
| CCV | B8 | 1.00 |  |  |  | 0.86 | 0.74 | 0.89 | 0.73 |
|  | B9 | 1.08 | 0.05 | 23.80 | *** | 0.92 | 0.85 |  |  |
|  | B10 | 0.90 | 0.05 | 19.71 | *** | 0.79 | 0.62 |  |  |

Note: ****p*＜0.001; Unstd, Unstandardized Factor Loadings; Std, Standardized Factor Loadings; SMC, Squared Multiple Correlation; CR, Composite Reliability; AVE, Average Variance Extracted.
